# Notes from Practice

**Published:** 1873-02

**Authors:** 


### ARTICLE II.

### *Notes from Practice.*

BY WESTMORELAND BROS., COLUMBUS, MISS.

Case 1.—*Diseased Antrum.* Mrs. T———, a lady o seemingly fine physical constitution, consulted us, complaining of a dull, deep-seated pain in the region of the antrum, saying that every few days there was a fullness, and after considerable pain she was relieved by a copious discharge, tinged with blood, from the nose. Upon examination a wisdom tooth was found in a very carious condition. It was removed, and the patient recoverd. We presume it yielded so readily because taken in time.

Case 2.—Mrs. F———, aged about 40, visited our office for the purpose of having two superior canines extracted, which had been broken off by another dentist. He had extracted all the others above for the purpose of inserting an artificial set. She desired gas, which we administered, extracting the teeth without much trouble. She complained also of a very severe pain, which we immediately recognized as that terrible disease of the antrum. She thought it was a case of neuralgia, caused by exposure, and hence had her teeth extracted. We immediately trephined with an instrument about the size of a goose-quill, through the first molar roots, and without any difficulty forced washes through. There was not much discharge at this time through the opening. We, of course, used the washes recommended in Harris' Principles and Practice, but not a particle of relief was obtained—the discharge growing worse all the time, irritating her throat, which we were obliged to cauterize. Large ulcers were visible. We finally consulted an intelligent surgeon, Dr. B———, who, after a description of the case, advised trephining again. An instrument about five-eighths of an inch was procured, and after making an incision extending from the canine around to the first molar, laying the wall of the antrum bare, an opening was made. A copious greenish discharge followed, and such an odor! One of us had to turn loose everything and run to the door to vomit. No chloroform was used, and persulphate of iron was used as a styptic. We used everything the doctor could suggest, and to-day she is no better than she was two years ago. We scraped the walls of the antrum. She refuses to submit to another operation.

Case 3.—Mrs. L——— called at our office with the same disease. Upon examination we found a very carious first bicuspid. We are ot the opinion that it was caused by an alveolar abscess, which she said pained her, swelling the parts, and finally went away—but since she was troubled with a discharge over her palate, trephined while the hole was open, (having extracted the tooth,) and gave her an ordinary syringe to wash with, recommending cold water. In a few days she returned no better—still discharging over the palate and through the nose. We procured a Davidson's India Rubber Syringe, and forced water through admirably; directed her to use it two or three months. She reported that she was perfectly well a few days ago.

Case 4.—Mrs. W———, a lady in delicate health, came thirty miles to have her teeth extracted for a set, according to the advice of her physician. She stated that she had one extracted about two years before, and came near bleeding to death. She was determined to have them extracted, saying, "they must come out if it kills me; there is no pleasure in life as it is." Accordingly on the third day after her arrival, we proceeded to extract two superior bicuspids, followed by no more bleeding than usual. On the next day (Thursday) we extracted two more on the opposite side; on Friday we extracted three—making seven, and persuaded her to wait until Monday. On Monday we extracted several, and Tuesday, all the remaining upper ones; none of them bleeding more than usual. On Tuesday night, on being summoned hurriedly to her bedside, it was found the blood was running from the first tooth extracted, a week before. We stopped it very readily by syringing it out with an injection containing a small quantity of carbolic acid, and applied persulphate of iron on a pledget of cotton.

To our surprise, the one on the opposite side commenced bleeding. We proceeded to arrest that in the same way, when shortly every one commenced bleeding. During the night she had a chill, and perhaps the fever caused this. We had considerable fears for her life. Learning that her general system was out of order, bowels costive, &c., we attended to them in the usual way, and at the same time guarded against a chill by giving 15 or 20 grains of quinine; administering an opiate to keep her quiet, and persisting in the use of persulphate of iron, succeeded in controlling it. She left for home in a few days, and after several months returned and had the lower teeth extracted, with which we had no trouble. She is wearing her set satisfactorily.

We have several other interesting cases, which we would be glad to lay before the readers of the JOURNAL, and may at some future time if this meets with approbation.

